# Bt GS57 Interaction With Gut Microbiota Accelerates *Spodoptera exigua* Mortality

**DOI:** 10.3389/fmicb.2022.835227

**Published:** 2022-03-23

**Authors:** Yazi Li, Dan Zhao, Han Wu, Yujie Ji, Zhaorui Liu, Xiaochang Guo, Wei Guo, Yang Bi

**Affiliations:** ^1^College of Plant Protection, Hebei Agricultural University, Baoding, China; ^2^Graduate School of Chinese Academy of Agricultural Sciences, Beijing, China; ^3^College of Bioscience and Resources Environment, Beijing University of Agriculture, Beijing, China

**Keywords:** Bt GS57, genomic feature, *Spodoptera exigua*, gut microbiota, diversity, dysbiosis

## Abstract

The Beet armyworm *Spodoptera exigua* (Lepidoptera: Noctuidae, Spodoptera) is an important global polyphagous pest. Pathogen infection could destroy the intestinal microbial homeostasis of insects, leading to the death of the host. However, the effect of the host intestinal microbial community on the insecticidal effect of *Bacillus thuringiensis* is rarely studied. In this study, the genome characteristics of Bt GS57 and the diversity and functions of the gut bacteria in *S. exigua* are investigated using crystal morphology, biological activity, and Illumina HiSeq high-throughput sequencing. The total size of the Bt GS57 genome is 6.17 Mbp with an average G + C content of 35.66%. Furthermore, the Bt GS57 genome contains six *cry* genes: *cry1Ca*, *cry1Da*, *cry2Ab*, *cry9Ea*, *cry1Ia*, and *cry1Aa*, and a vegetative insecticidal protein gene *vip3Aa*. The Bt GS57 strain can produce biconical crystals, mainly expressing 70 kDa and 130 kDa crystal proteins. The LC_50_ value of the Bt GS57 strain against the *S. exigua* larvae was 0.339 mg mL^–1^. Physiological and biochemical reactions showed that Bt GS57 belongs to *B.t.* var. *thuringiensis*. In addition, we found that *B. thuringiensis* can cause a dynamic change in the gut microbiota of *S. exigua*, with a significant reduction in bacterial diversity and a substantial increase in bacterial load. In turn, loss of gut microbiota significantly decreased the *B. thuringiensis* susceptibility of *S. exigua* larvae. Our findings reveal the vital contribution of the gut microbiota in *B. thuringiensis*-killing activity, providing new insights into the mechanisms of *B. thuringiensis* pathogenesis in insects.

## Introduction

*Bacillus thuringiensis* is a rod-shaped and Gram-positive bacterium, which produces a number of parasporal crystal proteins. *B. thuringiensis* shows different insecticidal activities against insect larvae without toxicity to animals (Nair et al., 2018). Therefore, it has been the most widely used biopesticide for several decades, owing to its toxicity toward a broad range of insect pests, such as *Helicoverpa armigera*, *Spodoptera exigua*, and *Plutella xylostella* (Lone et al., 2017; Su et al., 2017; Baragamaarachchi et al., 2019). Extensive planting of genetically engineered plants to produce Cry toxins has inhibited some major pests, reduced spraying of pesticides, enhanced natural enemy control of pests, and increased planting profit. However, these benefits have been eroded by the development of pest resistance (Gassmann, 2021). This resistance is a significant threat to the sustainability of *B. thuringiensis* biopesticide, thus reinforcing the need to find new *B. thuringiensis* strains and search for insect resistance mechanisms.

There are many *B. thuringiensis* genomes reported due to the rapid development of high-throughput sequencing technology; for instance, the *B. thuringiensi*s Al Hakam, *B. thuringiensis* YBT-1520, and *B. thuringiensis* HD73 (Challacombe et al., 2007; Liu et al., 2013; Zhu et al., 2015). Previous reports showed that *B. thuringiensis* strains have a genome size of 2.4–6.1 Mb and GC content of 32–36% (Carlson and Kolsto, 1993; Li et al., 2015; Wang et al., 2016; Cao et al., 2018). Illumina sequencing is characterized by high-throughput sequencing technology, with the ability to identify insecticidal genes quickly in combination with bioinformatics analysis (Qin et al., 2011), thus providing resources for transgenic and biopesticide research.

Insect gut microbiota has various functions, playing a crucial role in host physiology, especially in the development, metabolism, reproduction, maintenance of the insect immune system, and other life activities (Jones et al., 2013; Ramya et al., 2016; Douglas, 2018). Previous studies found that *Enterococcus* is beneficial to *Spodoptera littoralis* by protecting the insect host from pathogenic microorganisms toxins (Shao et al., 2017). Meanwhile, the genus *Bacillus* has been shown to help insects digest and absorb nutrients by producing lipases, amylases, and proteases (Ramya et al., 2016; Regode et al., 2016; Nguyen et al., 2018). Overall, gut microbiota in insects can affect the reproduction of pathogens and the development of insecticide resistance, which has become the focus of many insect gut microbiology studies. Hilbeck et al. (2018) found that gut bacteria can enhance the insecticidal activity of the Cry toxin protein by causing bacterial septicemia in *P. xylostella*. Li et al. (2021) found that Bt Cry1Ac toxin interacts with the gut bacteria of *P. xylostella* to accelerate larval death. Moreover, a pathogenic insect fungus called *Beauveria bassiana* has been shown to interact with the gut microbiota of mosquitoes, thereby accelerating mosquito mortality (Wei et al., 2017). Therefore, understanding the host gut microbial composition is essential for developing effective entomopathogen-based biological pesticides to manage pests in different environments.

Unlike fungi that cause host death mainly by penetrating the host integument and proliferating in the hemolymph, *B. thuringiensis* primarily interacts with protein receptors on the surface of insect gut cells through toxins, resulting in the formation of pores in the cell membrane and cell death (Mason et al., 2011; Lei et al., 2021). However, the interaction between the intestinal microbiota and the insect biocontrol bacteria *B. thuringiensis* remains unexplored. Prominent questions include: Can the intestinal microbiota protect the *S. exigua* from *B. thuringiensis* infection? Do gut microbes and *B. thuringiensis* interact, or do they function independently? When *B. thuringiensis* infects insects, does the intestinal microbiota become virulent? Understanding the tripartite interaction between *S. exigua*, gut bacteria, and *B. thuringiensis* biocontrol bacteria may provide new insights into the biocontrol bacteria-insect interactions. In addition, they may help develop new insect control strategies and disease intervention measures.

## Materials and Methods

### Insects Rearing and Antibiotic Treatment

*Spodoptera exigua* larvae were reared on an artificial diet under a controlled temperature of 27 ± 1°C, a photoperiod of 16-h light and 8-h dark, and relative humidity of 75 ± 10%, without exposure to any *B. thuringiensis* toxin. The rearing conditions of axenic *S. exigua* were the same as described previously (Broderick et al., 2006), except that the neonates were fed with a mixture of antibiotics (rifampicin, penicillin, streptomycin, and gentamicin) at a final concentration of 500 μg mL^–1^ until the second day of the 4th instar larvae.

### Insect Bioassays

The Bt GS57 strain was isolated from soil samples collected in Baoding, Hebei province, China, and identified with high toxicity against *S. exigua*. The Bt GS57 strain was inoculated in 1/2 LB medium and cultured at 30°C for 46 h until 70–90% of cell crystals were separated. Then, the spore crystal mixture was harvested and stored at −80°C. The spore crystal mixture was diluted to various concentrations (0.05, 0.1, 0.2, 0.4, 0.8, 1.6 mg mL^–1^) in sterile water and overlaid a 33 mm diameter plastic tube containing artificial diet. The control group was added the sterile water. After drying, the neonates of *S. exigua*, which had been starved for 2 h before the exposure to *B. thuringiensis*, were put into the plastic tube and reared under standard culture conditions (Zhang et al., 2017). Bioassays were repeated thrice for each treatment. Larval mortalities were quantified after 3 days, with 50% lethal concentration (LC_50_) values calculated using Probit analysis (SPSS, Chicago, IL, United States) (Finney, 1971).

### Bt GS57 Strain Phenotypic Characterization

The Bt GS57 was inoculated in LB media and kept at 30°C while shaking at 200 rpm. One milliliter of culture was sampled out from the conical flask repeatedly during the incubation period. In addition, the OD_600_ value was measured to observe the growth state of the cells to obtain microbial growth profiles.

We used a scanning electron microscope (SEM) and SDS-PAGE to characterize the Bt GS57 strain. The Bt GS57 spore-crystal mixtures were washed three times with ice-cold 0.01 M PBS. Then, the cells were harvested and fixed in 2.5% glutaraldehyde in 0.1 M PB for 2 h at 20°C. The samples were dehydrated in an ethanol graded series (30, 50, 70, 80, 90, 95%, and absolute ethanol) for 15 min each, before critical point drying. After fixation and dehydration, the samples were sprayed with gold using an ion sputtering equipment (IXRF, American) with a thickness of about 20–30 nm. Afterward, the material was examined and photographed in a HITACHI SU8100 (HITACHI, Tokyo, Japan) at a voltage of 3 kv. The SDS-PAGE of proteins was performed as described elsewhere (Nair et al., 2018). The molecular mass of proteins was determined using a high range protein molecular weight marker (10–250 kDa) obtained from the pre-stained protein ladder (Thermo Scientific, MA, United States).

### Physiological and Biochemical Characterization of Bt GS57

The colony shape and the color of the cells were observed by culturing on LB medium, NA medium, beef extract peptone agar medium, dextrose agar medium, and egg yolk agar medium at 30°C for 2–4 days. In addition, the VP reaction of Bt GS57, the production extracellular of amylase, lecithinase, and gelatinase, and the effect on esculin were measured. Finally, acid production was assessed using D-glucose, D-sucrose, D-cellobiose, and salicin media.

### Bt GS57 Genome Sequencing and Assembly

The Bt GS57 genome was sequenced using a PacBio RS II platform and Illumina HiSeq 4000 platform at the Beijing Genomics Institute (BGI, Shenzhen, China). PacBio subreads with a lengthless than 1 kb were removed during the analysis. The program Pbdagcon^1^ was used for self-correction. Draft genomic unitigs, which are uncontested fragments, were assembled against a high-quality corrected circular consensus sequence subreads set (Zuo et al., 2020). To improve the accuracy of the genome sequences, GATK^2^ and SOAP tool packages (SOAP2, SOAPsnp, SOAPindel) were used to make single-base corrections. To trace the presence of any plasmid, the filtered Illumina reads were mapped using SOAP to the bacterial plasmid database.

### Bt GS57 Genome Function Annotation

The best hit was subjected to Blast alignment tool for function annotation against ten databases: the VFDB (Virulence Factors of Pathogenic Bacteria), ARDB (Antibiotic Resistance Genes Database), CAZy (Carbohydrate-Active enZYmes Database), IPR Swiss-Prot, T3SS (Type III secretion system Effector protein), KEGG (Kyoto Encyclopedia of Genes and Genomes), COG (Clusters of Orthologous Groups), NR (Non-Redundant Protein Database databases), and GO (Gene Ontology). Function annotation was set at an E-value threshold of e < 1e^–5^.

### Phylogenetic Tree Analysis of Bt GS57

The 16S rDNA sequences of Bt GS57 were compared and aligned with other *Bacillus* species using ClustalX. The datasets includes 1,314 sites for 16S rDNA gene. Phylogenetic tree was generated with the MEGAX software, using neighbor-joining (NJ) method with Kimura 2-parameter model, which the gap opening penalty was 15, and gap extension penalty was 6.66 (Saitou and Nei, 1987). Branch supports were evaluated through the ultra-fast bootstrapping method with 1,000 replicates. The strains and their 16S rDNA corresponding to the GenBank accession numbers given below: *B. thuringiensis* BMB171 (CP001903.1); *B. thuringiensis* HD521 (CP010106); *B. thuringiensis* 97-27 (AE017355.1); *B. thuringiensis* YBT-020 (CP002508.1); *B. thuringiensis* Al Hakam (CP000485.1); *Bacillus cereus* ATCC 14579 (AE016877.1); *B. cereus* E33L (CP000001.1); *B. cereus* CCM 2010 (NR_115714.1); *B. cereus* IAM 12605 (NR_115526.1); *B. cereus* NBRC 15305 (NR_112630.1); *B. cereus* JCM 2152 (NR_113266.1); *Bacillus anthracis* SB1 (NR_118379.1); *B. anthracis* ATCC 14578 (NR_041248.1); *B. anthracis* SBS1 (NR_118536.1); *B. anthracis* CDC 684 (CP001215.1); *B. anthracis* Sterne (AE017225.1); *B. anthracis* Ames Ancestor (AE017334.2); *Bacillus megaterium* ATCC 14581 (NR_117473.1); *B. megaterium* IAM 13418 (NR_043401.1); *B. megaterium* DSM 32 (NR_118962.1); *Bacillus licheniformis* ATCC 14580 (NR_074923.1); *B. licheniformis* DSM 13 (NR_118996.1); *Bacillus atrophaeus* JCM 9070 (NR_024689.1); *B. atrophaeus* NBRC 15539 (NR_112723.1); *Bacillus cytotoxicus* NVH 391-98 (CP000764.1).

### *Spodoptera exigua* DNA Sample Preparation and Deep Sequencing

A 300 μLBt GS57 (4 mg mL^–1^) cell crystal mixture was spread on the diet surface to feed the 4th instar *S. exigua* larvae. The gut tissue (including contents) of the *S. exigua* larvae was extracted every 12 h and sequenced by Illumina MiSeq. Alterations of intestinal microbial diversity were analyzed at different time points (0, 12, 24, 48, and 72 h) of feeding Bt GS57. The *S. exigua* were surface-sterilized by immersion in 75% ethanol for 3 min and then rinsed three times in axenic RNase-free ddH_2_O. Afterward, they were dissected to separate the gut and the remainder of the body. The DNA was amplified by the 338F/806R primer (Table 1), which targets the V3 and V4 hypervariable region of the bacterial 16S rDNA gene. The 16S rDNA gene was sequenced on the Illumina MiSeq platform by Beijing Biomarker Technologies. The experiment was repeated three times.

**TABLE 1:** Primers used in this study.

Primer	(5′—3′) Primer sequence	Usage
338F	ACTCCTACGGGAGGCAGCA	PCR of V3 and V4 hypervariable regions of eubacterial 16S rRNA gene
806R	GGACTACHVGGGTWTCTAAT	
27F	AGAGTTTGATCCTGGCTCAG	PCR of eubacterial 16S rRNA gene
1492R	TACGGYTACCTTGTTACGACTT	
16SF	TCCTACGGGAGGCAGCAGT	qRT-PCR
16SR	GGACTACCAGGGTATCTAATCCTGTT	
*SeActin*-F	CTACCTCACGCCATTCTC	
*SeActin*-R	AACCTGAGTCTTTGTGTACCTCC	

### Quantification of Gut Bacteria by qRT-PCR

After Bt GS57 infection, samples were collected at four time points (0, 24, 48, and 72 h) based on the LC_50_ value in the 4th instar larvae of *S. exigua*. The bacterial DNA from *S. exigua* guts was extracted using TIANamp Genomic DNA Kit (Tiangen, Beijing, China), according to the manufacturer’s instructions. Bacterial quantitation by qRT-PCR was performed on genomic DNA using universal eubacteria primers to amplify the 16S ribosomal RNA (rRNA) fragments (Wei et al., 2017). The *Seactin* gene was used as an endogenous control (Table 1). Each reaction was performed in triplicate. The data were analyzed using the threshold cycle 2^–ΔΔCt^ method (Livak and Schmittgen, 2001).

### Isolation and Cultivation of Gut Microorganisms

To prepare the dissected guts, 4th instar larvae of *S. exigua* were surface sterilized in 75% ethanol for 3–5 min and then rinsed four times with PBS buffer. The guts were aseptically dissected in a plate containing axenic distilled water using sterilized forceps. It was then transferred to 300 μL axenic distilled water and homogenized for bacterial isolation (Wei et al., 2017). The *S. exigua* larvae were surface-sterilized, and the perfusion was collected. The gut homogenates were serially diluted 100,000 times with distilled water, plated onto LB agar plates, and then incubated for 2–3 days at 30°C, after which the colony forming units (CFUs) per plate were counted. The gut bacterial genomic DNA was extracted from collected samples using TIANamp Bacteria DNA Kit (Tiangen, Beijing, China), following the manufacturer’s instructions. The isolated bacteria were identified using 16S rRNA gene universal primers 27F/1492R (Table 1). The resulting PCR products were subjected to Sanger sequencing at Huada (Beijing, China). The sequenced bacterial strains were then identified via BLAST search.^3^ The phylogenetic tree was constructed using the neighbor-joining method using MEGAX software.

### The Role of Intestinal Microorganisms in the Insecticidal Activity of Bt GS57

The 300 μL Bt GS57 (4 mg mL^–1^) cell crystal mixture was added to the axenic diet. The axenic and non-axenic group of *S. exigua* was then compared. *B. cereus* HB1 and Enterococcus *mundtii* HB1 were cultured in LB medium at 37°C for 12 h until their OD_600_ reached 0.8. The bacterial culture was then pelleted by centrifugation (8,000 rpm for 6 min), washed twice in axenic ddH_2_O, and resuspended in axenic ddH_2_O to obtain 10^9^ cells per mL. Next, the 300 μL bacterial pellet was mixed with a 300 μL Bt GS57 (4 mg mL^–1^) cell crystal mixture. Lastly, bacterial suspension was added to an axenic diet, and then the activity was evaluated in sterilized *S. exigua*.

## Results

### Bt GS57 Strain Phenotypic Characterization

**FIGURE 1:**
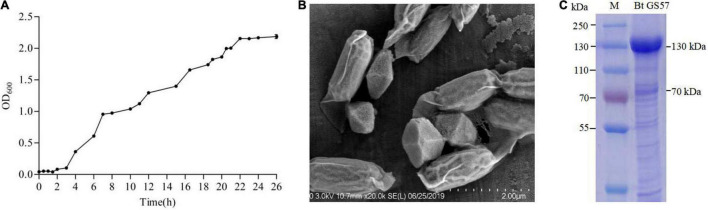
General characteristics of Bt GS57. **(A)** The growth curve of Bt GS57. **(B)** Scanning electron microscope (SEM) analysis of Bt GS57 spores and parasporal crystals. **(C)** SDS-PAGE analysis of spore-crystal suspension of Bt GS57: lane M, molecular mass standard; lane 1, Bt GS57.

The growth curve of Bt GS57 showed a 0–4 h delay and 5–20 h logarithmic growth periods. The stable growth had an OD_600_ between 2.046 and 2.155 (Figure 1A). The Bt GS57 can produce bipyramidal parasporal crystals during the stationary phase of its growth cycle (Figure 1B). The protein pattern of parasporal bodies as determined by SDS-PAGE consisted of two major bands of about 70–130 kDa, consistent with the its parasporal crystal gene (Figure 1C). The LC_50_ value of the Bt GS57 strain against *S. exigua* was 0.339 mg mL^–1^ (Table 2).

**TABLE 2:** Bioassay of Bt GS57 isolates against the neonates of *Spodoptera exigua.*

Bt isolate	Regression equation	Value of LC_50_ (mg mL^–1^)	95% Confidence interval (mg mL^–1^)
Bt GS57	*y* = 0.724 + 1.539x	0.339	0.178∼0.459

### Physiological and Biochemical Characterization of Bt GS57

Colonies of Bt GS57 strain were circular, white-colored, and rough in LB medium, NA medium, Beef extract peptone agar medium, Dextrose agar medium, and Egg yolk agar medium. The Bt GS57 utilized D-sucrose, D-cellobiose, and esculin, producing extracellular amylase, lecithinase, and gelatinase. Hydrolysis study showed that Bt GS57 can hydrolyze starch, gelatin, lecithin, and glycogen. The Bt GS57 does not produce pigments and membranes, and does not use glucose. Results of 10 physiological and biochemical reactions of *B.t.* var. *thuringiensis* were consistent; hence, we concluded that the Bt GS57 strain belongs to *B.t.* var. *thuringiensis*.

### Bt GS57 Genome Sequencing and Assembly

The Bt GS57 genome was sequenced using the Illumina (MiSeq) platform with paired-end reads sequencing. The total size of Bt GS57 genome was 6.17 Mbp with an average G + C content of 35.66%. In total, 6,597 genes, 6,532 coding sequences, 14 complete rRNAs, and 108 tRNAs were identified by PGAP analysis. Among 6,597 genes, 2,664 (40.38%) were smaller than 500 nt, 2,275 (34.49%) were between 500 nt and 1,000 nt, and 1,658 (25.13%) were longer than 1,000 nt, with an average gene length size of 757.66 nt (Supplementary Figure 1).

### Bt GS57 Genome Function Annotation

The 6,497 (98.48% of all distinct sequences) genes matched with the NR database (Supplementary Table 1), 2,674 (40.53%) to Swiss-Prot database (Supplementary Table 2), 3,324 (50.38%) to GO, 4,096 (62.08%) to COG, 3,041 (46.09%) to KEGG, 326 (4.94%) to VFDB, 53 (0.8%) to ARDB, 94 (1.42%) to CAZY, 5,106 (77.39%) to IPR, 755 (11.44%) to T3SS (Supplementary Figure 2). The summary statistics of the BLAST assignment are shown in Table 3 (Supplementary Table 3). GO enrichment of Bt GS57 gene function revealed that these genes include the “cellular component,” “biological process,” and “molecular function” (Supplementary Table 4) (Supplementary Figure 3) (Blake, 2013). To identify the metabolic pathways populated by these genes, 3,041 annotated genes were grouped into six categories: Cellular Processes, Environmental, Genetics, Human Disease, Metabolism, and Organismal System (Supplementary Table 5). The histogram obtained after the KEGG secondary classification statistics of each sample is shown in Supplementary Figure 4.

**TABLE 3:** Annotation of gene in venom apparatus.

Database	Number of annotated gene	Percentage of annotated gene
Nr	6,497	98.48%
VFDB	326	4.94%
ARDB	53	0.8%
CAZY	94	1.42%
IPR	5,106	77.39%
SWISSPROT	2,674	40.53%
COG	4,096	62.08%
GO	3,324	50.38%
KEGG	3,041	46.09%
T3SS	755	11.44%

*All 6,597 genes were annotated against Nr, VFDB, SWISSPROT, ARDB, CAZY, IPR, KEGG, T3SS, COG, and GO databases.*

### Bt GS57 Genome Properties

After assembly, the genome of Bt GS57 consisted of 4 replicons with a circular chromosome of 5,309,747 bp. The GC content of the circular chromosomes was 35.28% (Supplementary Figure 5). The three circular plasmids Bt GS57-1, Bt GS57-2 and Bt GS57-3 with a length of 76,132, 72,996, and 712,902 bp, respectively. The GC contents of the three plasmids ranged from 32.32 to 33.93% (Supplementary Figure 6). Genome annotation results showed that Bt GS57 has insecticidal genes *cry1Ca*, *cry1Da*, *cry2Ab*, *cry9Ea*, *cry1Ia*, *cry1Aa*, and *vip3Aa*.

### Phylogenetic Tree Analysis of Bt GS57

**FIGURE 2:**
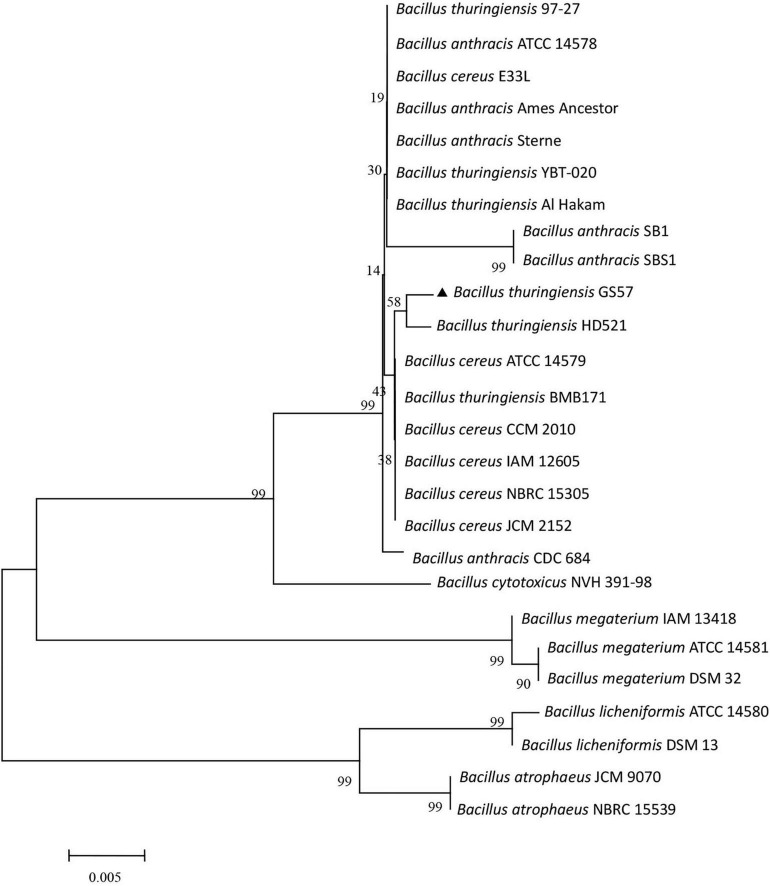
Phylogenetic tree based on 16S rDNA sequences Bt GS57 and *Bacillus cereus* sensu lato species group. The strains and their 16S rDNA corresponding to the GenBank accession numbers given below: *Bacillus thuringiensis* BMB171 (CP001903.1); *B. thuringiensis* HD521 (CP010106); *B. thuringiensis* 97-27 (AE017355.1); *B. thuringiensis* YBT-020 (CP002508.1); *B. thuringiensis* Al Hakam (CP000485.1); *B. cereus* ATCC 14579 (AE016877.1); *B. cereus* E33L (CP000001.1); *B. cereus* CCM 2010 (NR_115714.1); *B. cereus* IAM 12605 (NR_115526.1); *B. cereus* NBRC 15305 (NR_112630.1); *B. cereus* JCM 2152 (NR_113266.1); *Bacillus anthracis* SB1 (NR_118379.1); *B. anthracis* ATCC 14578 (NR_041248.1); *B. anthracis* SBS1 (NR_118536.1); *B. anthracis* CDC 684 (CP001215.1); *B. anthracis* Sterne (AE017225.1); *B. anthracis* Ames Ancestor (AE017334.2); *Bacillus megaterium* ATCC 14581 (NR_117473.1); *B. megaterium* IAM 13418 (NR_043401.1); *B. megaterium* DSM 32 (NR_118962.1); *Bacillus licheniformis* ATCC 14580 (NR_074923.1); *B. licheniformis* DSM 13 (NR_118996.1); *Bacillus atrophaeus* JCM 9070 (NR_024689.1); *B. atrophaeus* NBRC 15539 (NR_112723.1); *Bacillus cytotoxicus* NVH 391-98 (CP000764.1).

A phylogenetic tree was constructed to check whether the chimeric structure of the 16S rDNA gene affects species identification. Twenty-five *Bacillus* strains and Bt GS57 were chosen for phylogenetic analysis, showing more than 97% sequence similarity based on the basic local alignment search tool. The 16S rDNA sequence from *B. cytotoxicus* NVH 391-98 168 was selected as an outgroup. The phylogenetic tree illustrated that the Bt GS57 strain is closely related to *B. thuringiensis* serovar Indiana strain HD521 (Figure 2). However, the bootstraps value of the phylogenetic tree was very low because the 16S rDNA nucleotide sequence divergence of the chosen strains was also low, corroborating the previous study (Li et al., 2015). Ash et al. (1991) showed that 16S rDNA nucleotide sequences among *B. anthracis*, *B. thuringiensis*, and *B. cereus* were highly similar, exhibiting more than 99% sequence similarity.

### Bt GS57 Infection Causes Dysbiosis of the Gut Microbiota

**FIGURE 3:**
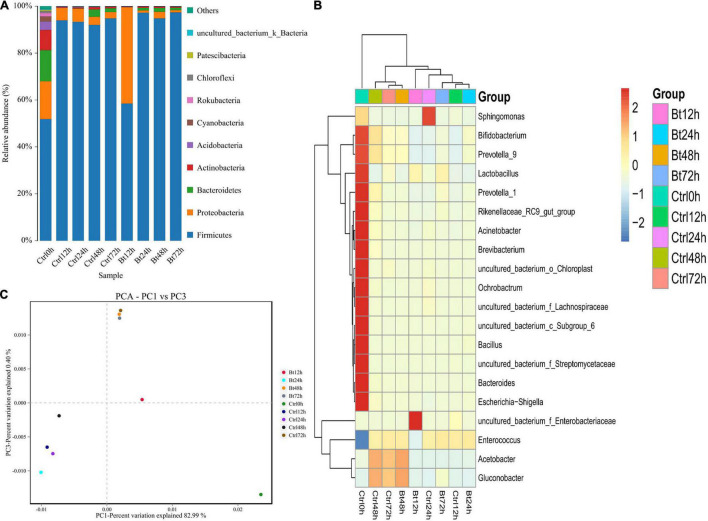
Bt GS57 infection changes in gut microbiota composition in *Spodoptera exigua*. **(A)** Histogram showing temporal changes, at the phylum level, in non-infected (Ctrl; ddH_2_O treatment as control) and Bt GS57-infected *S. exigua* (*n* = 45) over 72 h. **(B)** Heat map showing temporal changes, at the genus level, in Ctrl and Bt GS57-infected *S. exigua*. **(C)** Principal component analysis of microbial communities in different groups from Ctrl and Bt GS57-infected *S. exigua*.

Through deep sequencing of the 16S rRNA gene, we further assessed the gut bacteria’s dynamic composition and diversity in the non-infected *S. exigua* and the Bt GS57-infected *S. exigua* at 0, 12, 24, 48, and 72 h. In the non-infected *S. exigua*, the gut bacteria were diverse and dominated by bacteria of four phyla: Firmicutes, Proteobacteria, Bacterioidetes, and Actinobacteria (Figure 3A) (Supplementary Table 6). Firmicutes and Proteobacteria showed a dynamic change over time, possibly because of alterations in *S. exigua* physiology. The relative expression level of *Enterococcus* in the Bt GS57 treatment group was highest at 94.07%, and in the CK treatment group was lowest at 92.79% in 72 h (Figure 3B) (Supplementary Table 7).

Compared with non-infected *S. exigua*, Bt GS57 infection showed a reduced bacterial diversity (Simpson’s evenness, *P* < 0.05) (Table 4). Firmicutes predominated in Bt GS57-infected *S. exigua* at 24 h (Figure 3A). Furthermore, the composition and diversity of the gut bacterial population changed markedly in *S. exigua* after infection by Bt GS57, resulting in an almost exclusive colonization by one genus of Firmicutes–*Enterococcus*–in the Bt GS57-infected *S. exigua* (Figure 3B).

**TABLE 4:** The diversity index analyzed upon deep sequencing for each sample of gut microbiota of non-infected or Bt GS57-infected *Spodoptera exigua* at five time points.

		Ctrl					Bt GS57			
		0 h	12 h	24 h	48 h	72 h	12 h	24 h	48 h	72 h
Simpson	Average	5.36	0.51	0.71	0.96	0.72	1.19	0.47	0.69	0.56
	SD	1.12	0.20	0.29	0.49	0.16	0.19	0.17	0.02	0.14

The results of principal component analysis (PCA) showed that the gut microbial community composition differed between uninfected *S. exigua* and Bt GS57-infected *S. exigua* at 12, 24, and 48 h, while the gut microbial community composition was similar at 72 h (Figure 3C). These results indicate that Bt GS57 infection can alter gut microbial community composition.

### Effect of Bt GS57 on the Gut Bacterial Load of *Spodoptera exigua*

**FIGURE 4:**
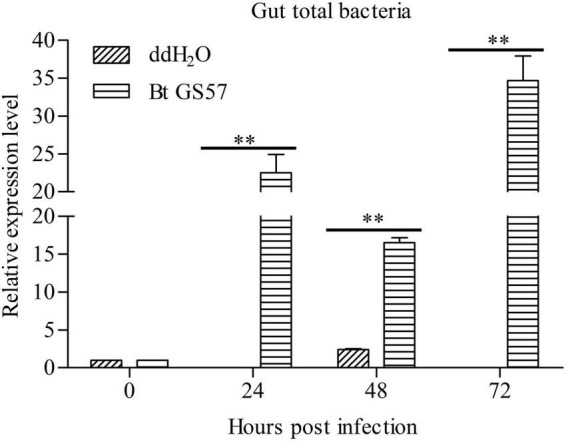
Effects of Bt GS57 infection on the gut bacterial load of *Spodoptera exigua*. The qPCR-based quantification of gut bacterial load in ddH_2_O and Bt GS57 treatment group of *S. exigua* (*n* = 6) at 0, 24, 48, and 72 h. Gene expression of each sample was normalized to that of *S. exigua* at time 0 (taken as 1). Data are representative of three independent experiments (mean + s.e.m.). The double asterisk indicates the significant difference determined by the student’s *t*-test, *P* < 0.01.

Next, we determined whether Bt GS57 infection affected the *S. exigua* gut microbiota by testing the bacterial loads in the gut of the *S. exigua* at 24, 48, and 72 h, through topical Bt GS57 infection. Results showed that the gut bacterial load was significantly upregulated in *S. exigua* following post-infection with Bt GS57 at different time intervals than non-infected controls treated with ddH_2_O. In addition, the qRT-PCR analysis of gut total bacterial load in infected *S. exigua* at 24 h showed that the relative expression level of bacterial load increased significantly, reaching a maximum level at 72 h (Figure 4).

### Detection of Axenic *Spodoptera exigua*

**FIGURE 5:**
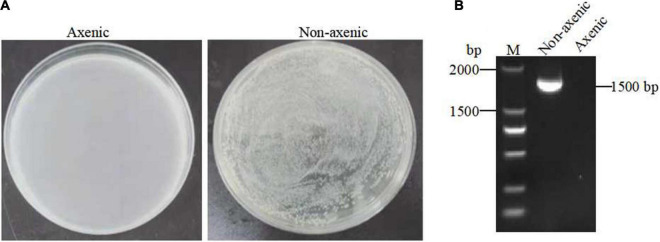
Generation of axenic *Spodoptera exigua*. **(A)** The efficacy of eliminating gut cultivable bacteria from non-axenic and axenic *S. exigua* (*n* = 3) gut homogenates on LB agar plates. **(B)** The efficacy of elimination of gut bacteria by performing PCR analysis on non-axenic and axenic *S. exigua* (*n* = 3) using 16S rRNA gene universal primers 27F/1492R.

To investigate the possible role of the gut microbiota in Bt GS57 in the *S. exigua*, axenic *S. exigua* was generated via treatment with oral antibiotics. The efficacy of elimination of gut bacteria was confirmed by plating gut homogenates onto LB agar plates and performing PCR analysis using bacterial 16S rDNA gene universal primers 27F/1492R, which detected unculturable microbes (e.g., obligate anaerobes) (Figures 5A,B and Table 1).

### Effect of Gut Microbiota on *Spodoptera exigua* Larval Susceptibility to Bt GS57

**FIGURE 6:**
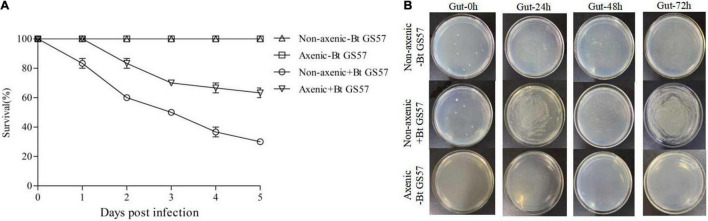
Effect of the gut microbiota on Bt GS57 in *Spodoptera exigua*. **(A)** Survival of axenic and non-axenic *S. exigua* (*n* = 45) following infection (+Bt GS57) or non-infectioun (−Bt GS57) with *Bacillus thuringiensis*. **(B)** The gut culturable bacterial load from axenic-Bt GS57, non-axenic + Bt GS57, and non-infected + Bt GS57 of *S. exigua* (*n* = 3) at 0, 24, 48, and 72 h post-Bt GS57 infection. Bacterial load was determined by plating the homogenate of *S. exigua* guts with 100,000 dilutions on LB agar plates.

An insect bioassay was conducted using the 4th instar larvae of *S. exigua* with and without gut microbiota. The biological activity test results of Bt GS57 showed that the mortality rate of axenic insects was significantly lower than that of non-axenic insects, and the mortality rate of non-axenic insects and axenic insects was 76.67 and 56.67% at 72 h, respectively (*P* < 0.01, *t*-test) (Figure 6). This result indicates that the gut microbiota accelerates the killing of *S. exigua* by Bt GS57.

### The Influence of *Enterococcus mundtii* HB1 or *Bacillus cereus* HB1 on the Insecticidal Activity of Bt GS57

**FIGURE 7:**
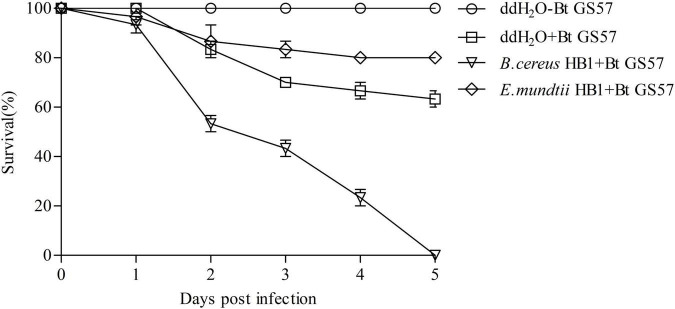
Survival of *Spodoptera exigua* fed *Bacillus cereus* and Enterococcus *mundtii* after infection with Bt GS57. Control *S. exigua* were fed ddH_2_O. Bacteria were introduced into the 4th instar larvae of *S. exigua* gut. Introduction of *B. cereus* (*B. cereus* + Bt GS57) significantly increased *S. exigua* susceptibility to Bt GS57 infection compared with the ddH_2_O + Bt GS57 treatment [log-rank (Mantel-Cox) test, *P* < 0.01]. The experiments were performed in three biological replicates. The log-rank test was used to assess the significance of differences between two survival curves using GraphPad Prism software.

Based on 16S rDNA gene sequence analysis, the two predominant cultivable bacteria showed high similarity with *E. mundtii* 15-1 and *B. cereus* FDAARGOS_797 and were named *E. mundtii* HB1 and *B. cereus* HB1, respectively (Supplementary Figure 7). To determine whether the proliferated *E. mundtii* HB1 and *B. cereus* HB1 contributed to the enhanced mortality speed, we reintroduced the overproliferating *B. cereus* HB1 and *E. mundtii* HB1 from the gut of Bt GS57-infected *S. exigua* into the gut of axenic *S. exigua*. We found that reintroducing *B. cereus* HB1 restored *S. exigua* susceptibility to Bt GS57 infection, while the reintroduction of *E. mundtii* HB1 reduced *S. exigua* susceptibility to Bt GS57 infection in comparison with ddH_2_O controls (Figure 7). At the same time, feeding *B. cereus* HB1 and *E. mundtii* HB1 alone did not cause the death of *S. exigua*.

## Discussion

*Bacillus thuringiensis*, as an environmentally friendly microbial pesticide, is already a useful alternative or supplement to synthetic chemical pesticide. Hence, it is a key source of genes for transgenic expression to provide pest resistance in plants (Ren et al., 2021). At present, *B. thuringiensis* pesticides have been widely used worldwide, accounting for about 90% of the microbial pesticide market in the United States (Chattopadhyay et al., 2004). However, the extensive use of *B. thuringiensis* insecticides and genetically modified plants has resulted in resistance to a large number of field pests. Furthermore, it has been reported that insects such as *P. xylostella*, *S. exigua*, *Helicoverpa zea*, *Spodoptera frugiperda*, and *Pectinophora gossypiella* have high resistance to the insecticidal protein from *B. thuringiensis* (Hernandez-Martinez et al., 2008; Tabashnik et al., 2008; Bagla, 2010; Storer et al., 2010; Naik et al., 2018; Xiong et al., 2021). Therefore, the search for new high insecticidal *B. thuringiensis* microbial insecticides, a clear composition of insecticidal genes of *B. thuringiensis* strain, and the expression of insecticidal proteins are of great significance for further understanding of *B. thuringiensis* insecticidal activity.

This study sequenced and characterized a novel *B. thuringiensis* strain isolated from soil. The complete genome of Bt GS57 exhibited some interesting features. Based on the next-generation sequencing of Illumina Hiseq2000 and *de novo* assembly, we found that Bt GS57 strain contains *cry1Ca*, *cry1Da*, *cry2Ab*, *cry9Ea*, *cry1Ia*, *cry1Aa, vip3Aa* gene, *chitinase*, and *zwittermicin.* Using genome sequencing to obtain more comprehensive information provides us with more gene resources for the biological control. It is of great significance in extending the scope of control and delaying the development of insecticide-resistance of the pest.

The gut microbiota contributes to the host’s health, and changes in the composition of the microbiota can cause disease (Pita et al., 2018; Li et al., 2020). The Cry toxin produced by *B. thuringiensis* creates pores in the membrane of the larval gut cells and disrupts insect gut microbial homeostasis (Portugal et al., 2017; Li et al., 2021). In this study, we evaluated the changes in the bacterial community of *S. exigua* after *B. thuringiensis* exposure. The significantly decreased Simpson’s index in *B. thuringiensis*-treated larvae suggested that they have a lower bacterial species richness than H_2_O-treated larvae (Table 4). At the phylum level, the results of our study were similar to other reports, namely the dominant bacterial phyla, such as Firmicutes and Proteobacteria (Gao et al., 2020). However, the dominant bacterial Firmicutes significantly increased while the quantity of Proteobacteria significantly reduced after feeding on *B. thuringiensis* (Figure 3). Therefore, changes in the composition of the gut microbiota caused by *B. thuringiensis* may adversely affect the physiological function of the insect. In addition, compared with the H_2_O-treated control group, the total amount of bacteria in the larvae exposed to *B. thuringiensis* increased significantly, further emphasizing the functional changes of intestinal bacteria after exposure to *B. thuringiensis* (Figure 6B). The qRT-PCR results showed that the gut bacterial load of *S. exigua* significantly increased in the Bt GS57 treatment group. Therefore, future studies should focus on the interaction between *B. thuringiensis* and *S. exigua* gut bacteria and their functions (Figure 4).

Many studies have shown that gut microbiota can enhance insecticide resistance (Kota et al., 2021; Hu et al., 2022). Inoculation of *Spodoptera litura* larvae with *E. mundtii* and *Enterococcus casseliflavus* significantly reduced susceptibility to methomyl (Hu et al., 2022). In *Cletus punctiger*, the gut commensal bacterium *Burkholderia* can degrade organophosphate compounds and remarkably increase the resistance against fenitrothion (Kota et al., 2021). A previous study has suggested that axenic *Lepidopteran* insects are not sensitive to *B. thuringiensis*, where inoculation of midgut bacteria can restore *B. thuringiensis* pathogenicity (Paramasiva et al., 2014). This is supported by previous work of Paramasiva et al. (2014), showing that antibiotics eliminated from gut microflora influenced the toxicity of *B. thuringiensis* against *H. armigera*. Wei et al. (2017) also confirmed that *B. bassiana* interacts with mosquito gut microorganisms to accelerate mosquito death. In this study, we have investigated and evaluated the role of the gut microbiota in the interaction of the Bt GS57 with *S. exigua*. Our findings indicate that Bt GS57 interacts with the gut microbiota to promote *S. exigua* death (Figure 6A). Reintroduction of gut *B. cereus* HB1 and *E. mundtii* HB1 into axenic *S. exigua* revealed that *B. cereus* HB1 can significantly enhance the susceptibility of the *S. exigua* to Bt GS57, while *E. mundtii* HB1 can inhibit the vulnerability of the *S. exigua* to Bt GS57 (Figure 7). Raymond et al. (2008) found that antibiotics secreted by *B. cereus* can reduce the abundance of symbiotic gut microbiota and promote the infection of *B. thuringiensis* in the co-infection experiment of *B. cereus* and *B. thuringiensis*. However, Shao et al. (2017) pointed out that the *E. mundtii*, a commensal gut bacteria, produces antimicrobial peptides to kill foreign pathogens effectively, thereby suppressing the mortality of *S. exigua*. These may result from the interaction between *B. thuringiensis* and the gut bacteria of *S. exigua*.

In conclusion, this study carried out a novel analysis of the wild Bt GS57 strain and identified the insecticidal gene at the genomic level. Furthermore, the insect bioassay confirmed that the gut microbiota accelerates killing of *S. exigua* by Bt GS57. Related research provides reference data for elucidating the functional characteristics of the Bt GS57 strain, further understanding the interaction of *B. thuringiensis*-insect-microbiota, and exploring its insecticidal potential.

## Data Availability Statement

The datasets presented in this study can be found in online repositories. The names of the repository/repositories and accession number(s) can be found in the article/Supplementary Material.

## Author Contributions

YL, DZ, and WG conceived the research. YL, DZ, HW, and YJ conducted the experiments. DZ and WG contributed the material. ZL, XG, and YB analyzed the data and conducted the statistical analyses. YL wrote the manuscript. WG secured the funding. All authors read and approved the manuscript.

## Conflict of Interest

The authors declare that the research was conducted in the absence of any commercial or financial relationships that could be construed as a potential conflict of interest.

## Publisher’s Note

All claims expressed in this article are solely those of the authors and do not necessarily represent those of their affiliated organizations, or those of the publisher, the editors and the reviewers. Any product that may be evaluated in this article, or claim that may be made by its manufacturer, is not guaranteed or endorsed by the publisher.

## Footnotes

1
https://tracker.debian.org/pkg/pbdagcon

2
https://www.broadinstitute.org/gatk/

3
https://blast.ncbi.nlm.nih.gov/Blast.cgi
