# Supplementary material for: Bt GS57 Interaction With Gut Microbiota Accelerates Spodoptera exigua Mortality
Source: Front Microbiol. 2022 Mar 23;13:835227. doi: 10.3389/fmicb.2022.835227 (PMC8989089; doi:10.3389/fmicb.2022.835227)
Supplement: Supplementary file 1 [file Data_Sheet_1.doc]

**Supplementary Materials**

**Bt GS57 interacts with the gut microbiota to accelerate *Spodoptera exigua* mortality**

Yazi Li^1†^, Dan Zhao^1†^, Han Wu^1^, Yujie Ji^2^, Zhaorui Liu^1^, Xiaochang Guo^1^, Wei Guo^1,2#^ and Yang Bi^3^

^1^ College of Plant Protection, Hebei Agricultural University, Baoding, 071000, China.

^2^ Graduate School of Chinese Academy of Agricultural Sciences, Beijing, 100081, China.

^3^ College of Bioscience and Resources Environment, Beijing University of Agriculture, Beijing, 102206, China.

^#^ correspondence to guowei@hebau.edu.cn.

^†^ These authors contributed equally to this work.

**This file includes:**

Supplementary Figures 1-7


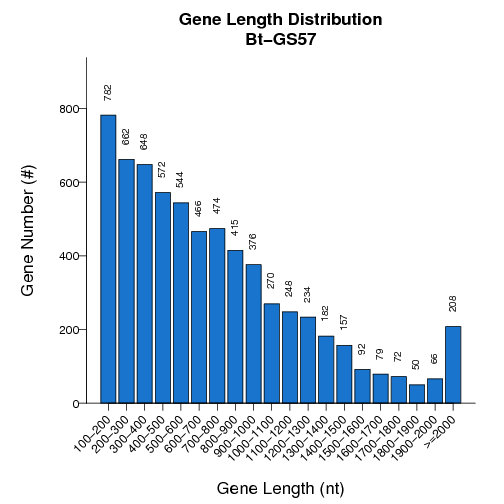


**Supplementary Figure 1 Gene length distribution.** The X-axis indicates the gene length and Y-axis indicates the number of gene of each gene length.

### **
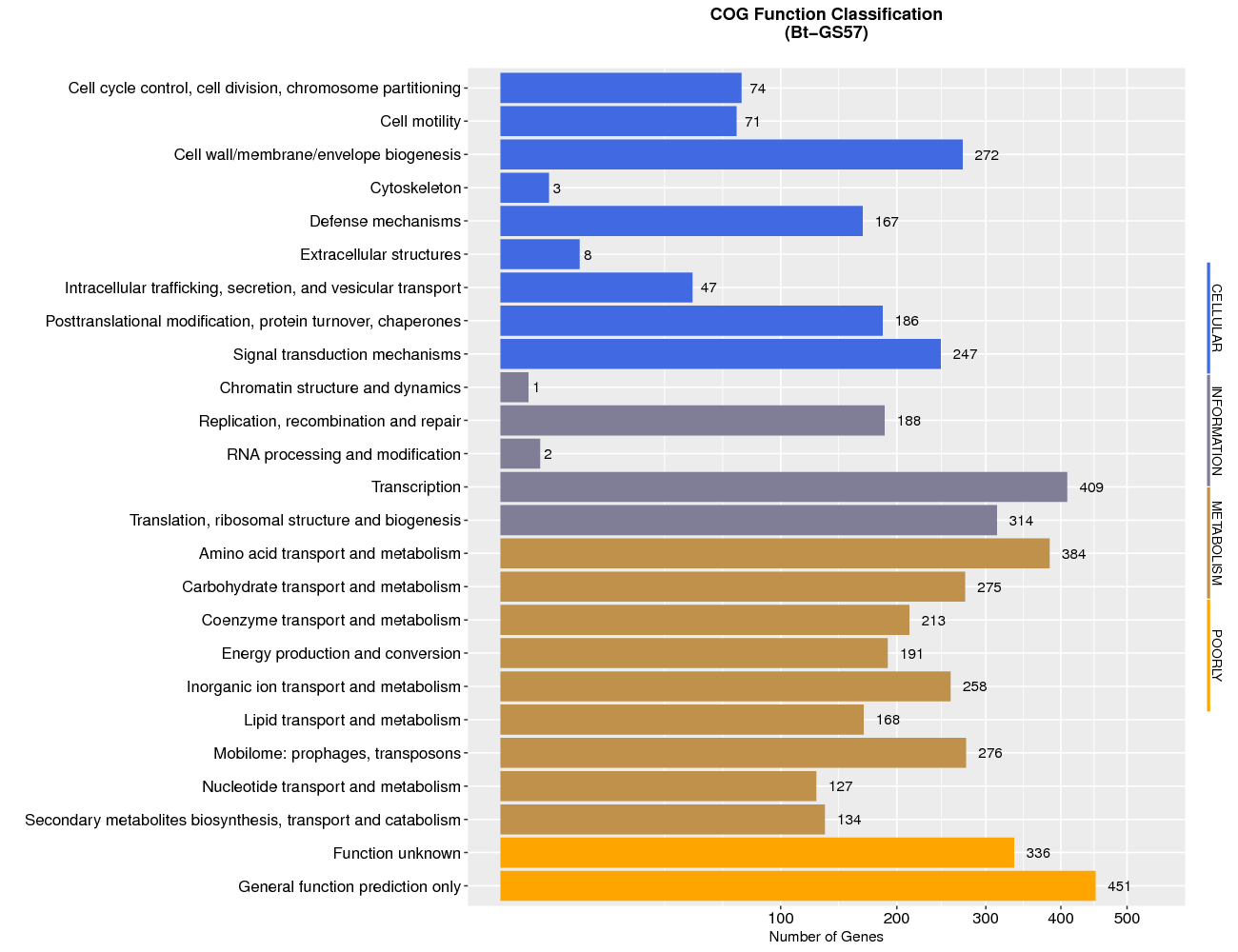
**

### **Supplementary Figure 2 The distribution of genes function against COG database.** The X-axis indicates the number of genes and Y-axis indicates the comment entry.


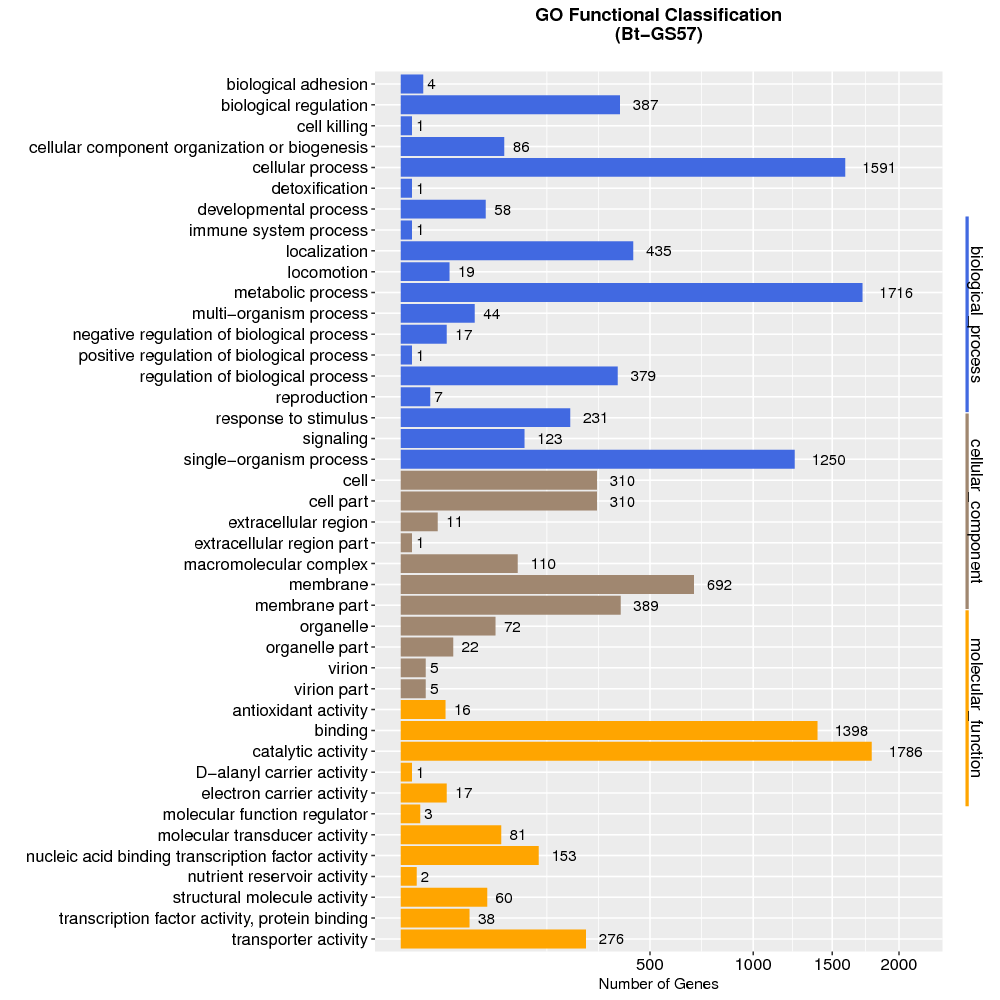


### **Supplementary Figure 3 The distribution of genes function classification against GO database.** 3,324 genes were divided into three categories: "cellular component", "biological process" and "molecular function". The X-axis indicates the number of genes and Y-axis indicates the comment entry.


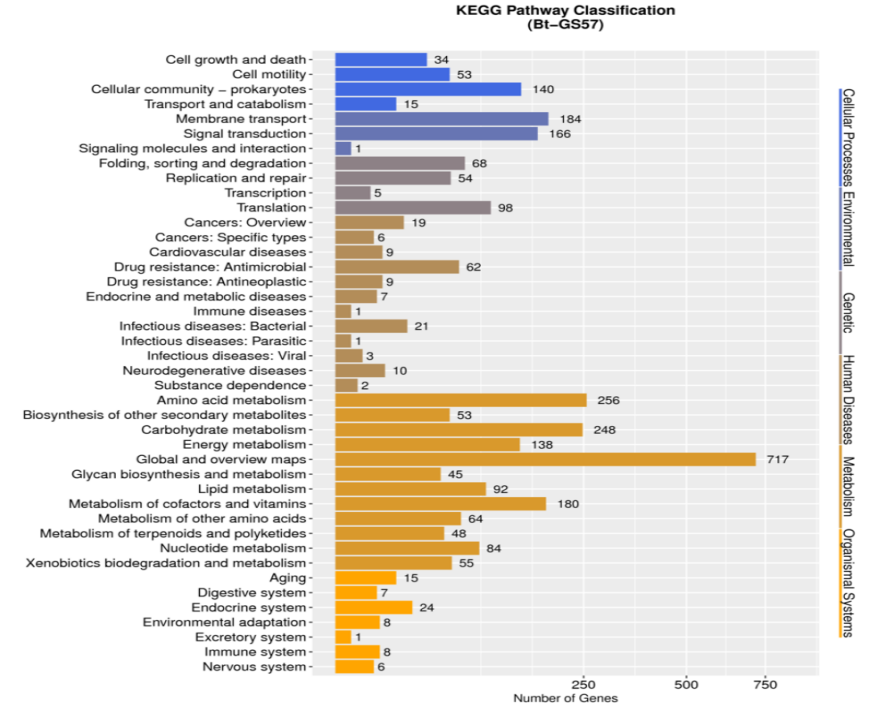


### **Supplementary Figure 4 KEGG categories of annotated genes function.** 3,041 genes were divided into six categories: Cellular Processes, Environmental, Genetic, Human Disease, Metabolism and Organismal System. The X-axis indicates the number of genes and Y-axis indicates the comment entry.


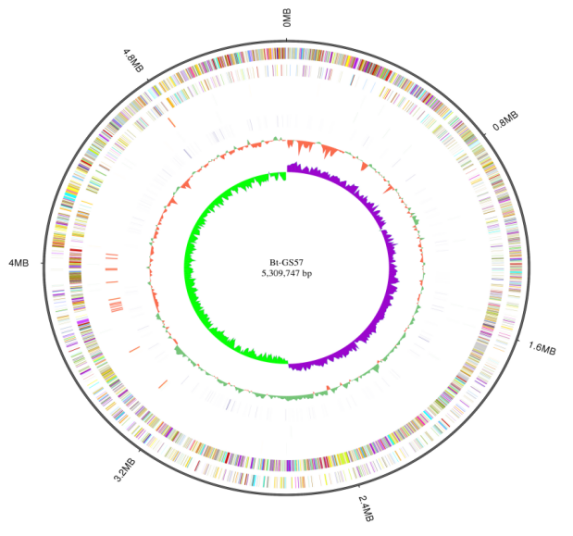


**Supplementary Figure 5 Circular representation of chromosome of Bt GS57 performing relevant genome features.** From outside to inner: Genome Size，Forward Strand Gene, colored according to cluster of orthologous groups (COG) classification, Reverse Strand Gene, colored according to cluster of orthologous groups (COG) classification, Forward Strand ncRNA, Reverse Strand ncRNA, repeat, GC, GC-SKEW.


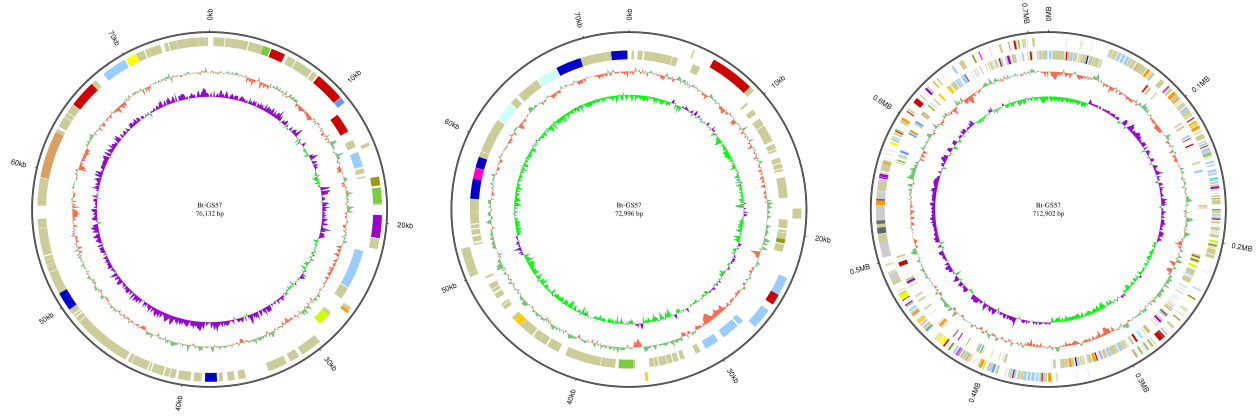


**Supplementary Figure 6 Circular representation of plasmide of Bt GS57 performing relevant genome features.** The plasmide 1-3 from outside to inner: Genome Size, Forward Strand Gene, colored according to cluster of orthologous groups (COG) classification, Reverse Strand Gene, colored according to cluster of orthologous groups (COG) classification, GC, GC-SKEW.


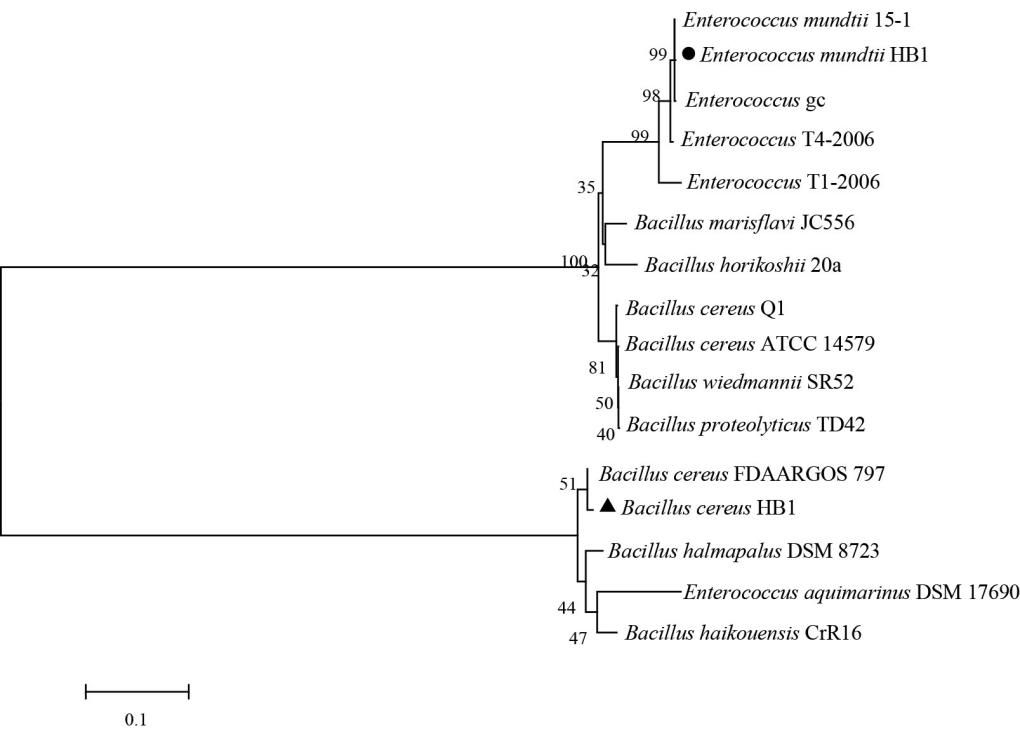


**Supplementary Figure 7 Phylogenetic tree based on 16S rDNA sequences predominant cultivable bacterium with 14 related bacteria.** The strains and their 16S rDNA corresponding to the GenBank accession numbers given below: *E. mundtii* 15-1 (NZ_AP019810.1); *E. aquimarinus* DSM 17690 (NZ_JXKD01000005.1); *Enterococcus* gc (DQ469876.1); *Enterococcus* T1-2006 (DQ462332.1); *Enterococcus* T4-2006 (DQ462331.1); *B. cereus* FDAARGOS_797 (NZ_CP053931.1); *B. wiedmannii* SR52 (NZ_CP032365.1); *B. proteolyticus* TD42 (NZ_MACH01000033.1); *B. horikoshii* 20a (NZ_CP020880.1); *B. cereus* Q1 (CP000227.1); *B. cereus* ATCC 14579 (NR_074540.1); *B. marisflavi* JC556 (LS974830.1); *B. halmapalus* DSM 8723 (NR_026144.1); *B. haikouensis* CrR16 (NZ_JABMCR010000501.1).
